# Teaching beliefs and wellbeing among primary school teachers: a moderated mediation of work engagement and career adaptability

**DOI:** 10.3389/fpsyg.2026.1835767

**Published:** 2026-05-28

**Authors:** Jingjing Wang, Xue Xu

**Affiliations:** School of Education Science, Hanshan Normal University, Chaozhou, Guangdong, China

**Keywords:** career adaptability, job demands-resources model, moderated mediation model, primary school teachers, teaching beliefs, wellbeing, work engagement

## Abstract

**Objective:**

Drawing on an integrative analytical framework drawing on the Job Demands–Resources (JD-R) model and Career Construction Theory, this study investigated the conditional indirect mechanism underlying the association between teaching beliefs and teacher wellbeing, with a particular focus on the mediating role of work engagement and the moderating effect of career adaptability.

**Methods:**

A questionnaire survey was administered to 561 primary school teachers in Guangdong Province, China, using the Teaching Beliefs Scale (TBS), the Career Adapt-Abilities Scale (CAAS), the Utrecht Work Engagement Scale (UWES-17), and the General wellbeing Schedule (GWB). A moderated mediation model was tested with Statistical Package for the Social Sciences (SPSS) 26.0 and the PROCESS macro (Model 7).

**Results:**

The main findings were as follows: (1) Teaching beliefs, career adaptability, work engagement, and wellbeing were all significantly and positively intercorrelated (*r* = 0.376–0.696, *p*<*0*.001); (2) work engagement partially mediated the cross-sectional relationship between teaching beliefs and wellbeing; (3) career adaptability significantly and positively moderated the cross-sectional relationship between teaching beliefs and work engagement (*B* = 0.123, *p*<*0*.001); (4) the index of moderated mediation was statistically significant [Index = 0.051, 95% CI (0.022, 0.079)]; Johnson–Neyman analysis indicated that approximately 43.7% of the teachers fell within the region of non-significance (raw career adaptability score < 4.11).

**Conclusion:**

These findings indicate that teaching beliefs are conditionally and indirectly associated with wellbeing via work engagement, and this pathway is systematically moderated by career adaptability. For teachers with insufficient career adaptability, prioritizing the enhancement of meta-resource levels may serve as a critical condition for the beliefs–engagement–wellbeing associational pathway to become statistically detectable.

## 1 Introduction

Teacher wellbeing is not only critical to individual teachers' physical and mental health and long-term career sustainability, but also constitutes a key prerequisite for safeguarding educational quality and fostering students' holistic development (Hascher and Waber, 2021). In recent years, the ongoing deepening of educational policy reforms has subjected teachers to multiple pressures, including role transformation and changes in working practices (Kun and Gadanecz, 2022). Within the Chinese context, primary school teachers are additionally exposed to sustained multidimensional stressors (Sun et al., 2022; Liao et al., 2023), encompassing student academic performance, home-school communication, and professional title evaluation. Against this backdrop, systematically elucidating the key determinants of primary school teachers' wellbeing and the mechanisms through which these determinants operate carries important theoretical value and practical significance for developing evidence-based teacher support policies and promoting sustainable teacher development.

### 1.1 Teaching beliefs and teacher wellbeing

Social cognitive theory (Bandura, 1997) posits that beliefs constitute a core mechanism regulating individual behavior and emotional experience. Teaching beliefs refer to teachers' relatively stable cognitive and evaluative dispositions regarding the nature of teaching, student learning, the properties of knowledge, and their own professional roles (Pajares, 1992), and they function as a “mental filter” (Fives and Buehl, 2012). Primary school teachers who hold positive teaching beliefs tend to place a higher value on their professional roles and to possess greater confidence in their teaching efficacy, and these cognitive resources collectively form an important psychological foundation for wellbeing.

Existing empirical research has consistently supported a positive relationship between teaching beliefs and teacher wellbeing. Longitudinal studies have found that teachers holding student-centered beliefs report higher job satisfaction and lower emotional exhaustion (Voss and Kunter, 2020). Dreer (2021) further demonstrated that positive teaching beliefs enhance wellbeing by promoting positive emotional experiences, an effect that remained robust after controlling for work-related stress. Research focusing specifically on primary school teachers has shown that teaching beliefs are significantly and positively associated with self-efficacy, work engagement, and instructional behavior (Reppa et al., 2023; Luján, 2021; Li et al., 2024), and that a growth-oriented teaching mindset positively predicts positive affect and professional satisfaction (Nalipay et al., 2022, 2019).

***Hypothesis 1:*** *teaching beliefs are significantly and positively associated with primary school teachers' wellbeing*.

### 1.2 The mediating role of work engagement

Work engagement is a positive, work-related psychological state characterized by vigor, dedication, and absorption (Schaufeli et al., 2002), and represents the core outcome variable in the motivational pathway of the JD-R model. Meta-analytic evidence demonstrates that cognitive personal resources such as beliefs and values are particularly stable predictors of work engagement (Lesener et al., 2020). Teachers who hold constructivist or student-centered teaching beliefs are more inclined to perceive teaching as meaningful professional practice, thereby being more likely to experience heightened intrinsic motivation and a “resource gain spiral” (Bakker and Demerouti, 2017).

The mediating role of work engagement has been well-established in educational contexts. Hakanen et al. (2006) found that job resources promote teacher wellbeing via work engagement; Granziera et al. (2020) further demonstrated that personal belief resources can exert an indirect influence on wellbeing through work engagement, a pathway that remained robust in longitudinal data. Research on primary and secondary school teachers has also found that work engagement plays a significant mediating role between work values and teacher wellbeing (Shao et al., 2025; Feng et al., 2024).

***Hypothesis 2:*** *work engagement partially mediates the relationship between teaching beliefs and primary school teachers' wellbeing*.

### 1.3 The moderating role of career adaptability

Career Construction Theory (Savickas, 2013) defines career adaptability as an individual's collection of psychosocial resources for coping with vocational tasks, occupational transitions, and work-related challenges, conceptualized across four dimensions: concern, control, curiosity, and confidence (Savickas and Porfeli, 2012). Meta-analytic findings indicate that career adaptability is moderately and positively correlated with job satisfaction and work engagement (Rudolph et al., 2017).

Career Construction Theory characterizes career adaptability as a meta-resource associated with individuals' capacity to mobilize, integrate, and deploy specific vocational resources such as teaching beliefs (Haenggli and Hirschi, 2020). Primary school teachers with high career adaptability can align their positive teaching beliefs with a coherent career plan, translating belief intentions into purposeful engagement behaviors. In contrast, teachers with low career adaptability may experience a “belief–action gap.” Conservation of Resources Theory (Hobfoll, 2001) further predicts that under conditions of low career adaptability, the meta-capacities required to externalize beliefs into high engagement are insufficient, thereby limiting the conversion efficiency of the beliefs–work engagement link (Haenggli and Hirschi, 2020).

***Hypothesis 3:*** *career adaptability significantly and positively moderates the relationship between teaching beliefs and work engagement*.

***Hypothesis 4:*** *the index of moderated mediation is statistically significant*.

**Figure 1:**
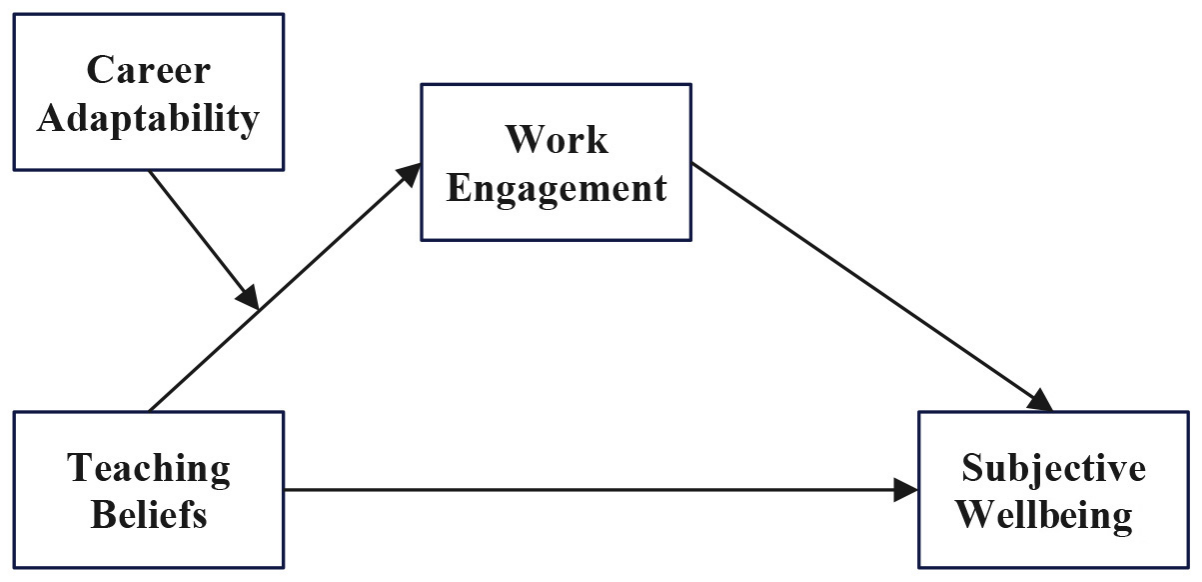
Conceptual model of the study.

Relative to the existing literature, the present study makes incremental contributions in three respects. First, at the theoretical level, it proposes and tests an integrative analytical framework that uses Career Construction Theory to address a specific explanatory gap within the JD-R model—namely, the model's limited specification of the conditions under which personal resources (teaching beliefs) translate into motivational outcomes (work engagement). The new theoretical mechanism proposed herein is that career adaptability functions as a meta-resource that regulates the resource-conversion efficiency of the JD-R motivational pathway; the added value thus lies not in the additive sum of two frameworks but in the identification of a meta-resource moderating mechanism that is not native to the JD-R model and that Career Construction Theory uniquely supplies. Second, at the mechanistic level, it identifies and empirically validates conditional heterogeneity in the mediated effect, thereby delineating the boundary conditions of the beliefs → engagement → wellbeing pathway. Third, at the contextual and methodological level, it employs the Johnson–Neyman technique to precisely demarcate the region of significance for the moderating effect, providing new cross-cultural empirical evidence for the applicability of Career Construction Theory in non-Western primary education contexts. The complete conceptual model of this study is presented in Figure 1.

## 2 Methods

### 2.1 Ethics statement

This study was approved by the Ethics Committee of Hanshan Normal University (Approval Code: 2025092406; Approval Date: September 24, 2025) and was conducted in accordance with the Declaration of Helsinki. All participants provided electronic informed consent prior to completing the questionnaire. Data collection was anonymous, and all authors declared no conflicts of interest.

### 2.2 Participants

Using convenience sampling, 600 questionnaires were distributed via an online survey platform to primary school teachers in multiple cities and districts of Guangdong Province, China, in February 2026. After excluding invalid responses, 561 valid questionnaires were retained (effective response rate: 93.5%). With reference to the recommendations of Fritz and MacKinnon (2007) and Preacher et al. (2007), a minimum of 500 participants is required to detect moderated mediation effects under medium effect size conditions; the present sample thus satisfies statistical power requirements.

Demographic characteristics of the sample were as follows: 406 female (72.4%) and 155 male (27.6%), a gender distribution largely consistent with the national gender structure of primary and secondary school teachers in China (Ministry of Education, 2023). In terms of age, 35 participants (6.2%) were below 30 years old, 231 (41.2%) were between 31 and 40, 247 (44.0%) were between 41 and 50, and 48 (8.6%) were between 51 and 60, with a mean age of 40.2 years (*SD* = 7.8). Mean teaching experience was 15.6 years (*SD* = 8.3). Regarding educational attainment, 112 participants (20.0%) held junior college degrees and 449 (80.0%) held bachelor's degrees or above. With respect to professional rank, 53 (9.4%) had no rank, 126 (22.5%) held junior rank, 338 (60.3%) held intermediate rank, and 44 (7.8%) held senior rank.

### 2.3 Measures

(1) Teaching Beliefs Scale (TBS). The Teaching Beliefs Scale for Primary School Teachers developed by Wang et al. (2022) was used. Transparency disclosure: this scale was developed by the first author of the present study. To mitigate potential self-serving bias, the following should be noted: (a) the scale was originally published and peer-reviewed independently of the present study (Journal of Hanshan Normal University, 2022); (b) the current CFA was conducted on a new independent sample (*N* = 561) rather than the original development sample; and (c) the scale has been cited and applied in subsequent research by independent teams. Readers should nonetheless be aware that use of a self-developed instrument constitutes a limitation, and the findings regarding teaching beliefs should be interpreted with corresponding caution (see Section 4.5). The scale comprises 27 items (e.g., “Student management should be approached from the perspective of caring for and respecting students”) across four dimensions: assistance in learning, role perception, relationship optimization, and self-efficacy. Items are rated on a 5-point Likert scale (1 = strongly disagree, 5 = strongly agree). Scale scores were computed as the mean of all items within each dimension, with higher mean scores indicating more positive teaching beliefs. In the present study, CFA confirmed a well-fitting four-factor model (χ^2^*/*df = 2.34, *Comparative Fit Index (CFI)* = 0.91, *Tucker–Lewis Index (TLI)* = 0.90, *Root Mean Square Error of Approximation (RMSEA)* = 0.049, *Standardized Root Mean Square Residual (SRMR)* = 0.052), Cronbach's α = 0.817, *CR* = 0.838.

(2) Career Adapt-Abilities Scale (CAAS). The Chinese version of the CAAS developed by Savickas and Porfeli (2012) and revised by Hou et al. (2012) was used. The scale consists of 24 items (e.g., “I often think about what my future will be like”) across four subscales: concern, control, curiosity, and confidence, rated on a 5-point scale (1 = not strong, 5 = strongest). Scale scores were computed as the mean of all items, with higher scores reflecting greater career adaptability. CFA yielded good fit (χ^2^*/*df = 2.89, *CFI* = 0.94, *TLI* = 0.93, *RMSEA* = 0.058, *SRMR* = 0.041), α = 0.956, *CR* =0.958.

(3) Utrecht Work Engagement Scale (UWES-17). The Chinese version of the UWES-17 developed by Schaufeli et al. (2002) and validated in Chinese samples was used. Note: Schaufeli et al. (2002) reported the original two-sample CFA validation of the UWES; the Chinese adaptation used in the present study followed the localization procedures established by Zhang and Gan (2005), who confirmed factorial validity and reliability of the Chinese UWES-17 in a large teacher sample. The scale comprises 17 items (e.g., “At my work, I feel that I am bursting with energy”) across three subscales: vigor, dedication, and absorption, rated on a 7-point scale (0 = never, 6 = always). Scale scores were computed as the mean of all items. CFA yielded good fit (χ^2^*/*df = 2.67, *CFI* = 0.95, *TLI* = 0.94, *RMSEA* = 0.055, *SRMR* = 0.038), α = 0.959, *CR* = 0.959.

(4) General wellbeing Schedule (GWB). The GWB developed by Fazio (1977) was used. The scale consists of 18 items (e.g., “How have you been feeling in general?”), with response formats varying by item (including both Likert-type and bipolar ratings). Scale scores were computed as the sum of item scores, with higher scores indicating greater wellbeing. Single-factor CFA fit indices were (χ^2^*/*df = 3.12, *CFI* = 0.92, *TLI* = 0.90, *RMSEA* = 0.062, *SRMR* = 0.048), α = 0.834, *CR* = 0.899.

### 2.4 Data analysis

SPSS 26.0 (IBM Corp, 2019) was used for descriptive statistics, correlation analysis, and tests for common method bias; Mplus 8.3 (Muthén and Muthén, 1998–2017) was used for confirmatory factor analysis (*CFA*); and the PROCESS macro (Model 7; Hayes, 2022) was used to test the moderated mediation model, with bias-corrected bootstrapping (5,000 resamples) employed to estimate indirect effects and their 95% confidence intervals. Simple slope analysis and the Johnson–Neyman technique were used to determine the region of significance for the moderating effect. These variables were selected on theoretical and empirical grounds: gender was included because research on Chinese teachers has examined gender differences in occupational stress and mental health, highlighting the importance of testing gendered patterns of occupational wellbeing in this context (Ji et al., 2021). Age and teaching experience were included as proxies for career stage, given meta-analytic evidence that career adaptability is linked to job stress, engagement, and other work outcomes across demographic groups, and that age and education relate to adaptability resources over time (Rudolph et al., 2017). Educational attainment and professional rank were included because they index access to professional resources, development opportunities, and recognition within the Chinese educational system, which have been linked to shape teachers' workloads, duties, and opportunities and thus their occupational wellbeing (Gao et al., 2024). All continuous variables were standardized prior to analysis. Effect size indices *R*^2^, Δ*R*^2^, Cohen's *f*^2^ (Cohen, 1988), and κ^2^ (Preacher and Kelley, 2011) are reported.

## 3 Results

### 3.1 CMB

Three complementary approaches were employed to assess CMB. (1) Harman's single-factor test: an unrotated exploratory factor analysis of all 86 items revealed that the first factor accounted for 27.84% of the total variance, falling below the conventional 40% threshold. (2) Common latent factor method: after incorporating a latent method factor, *CFI* improved by only 0.012 and *RMSEA* decreased by only 0.004, with a mean standardized loading of 0.11 (range: 0.02–0.23) across items on the method factor. (3) The four-factor model significantly outperformed the single-factor model (Δχ^2^ = 2847.36, Δdf = 6, *p* < 0.001). Collectively, these results provide preliminary evidence that severe common method bias is unlikely. However, several important caveats are warranted. First, Harman's single-factor test has been widely criticized as a *post hoc* diagnostic of limited power (Podsakoff et al., 2003): passing the 40% threshold indicates that a single general factor cannot account for all covariance, but it does not eliminate the possibility of moderate method-induced inflation in observed correlations. Second, the common latent factor method's small mean loading (0.11) is reassuring, but this technique's effectiveness depends on whether the latent method factor adequately captures the actual sources of shared variance, which may vary across item formats and scales. Third, because all four constructs were assessed via self-report at a single time point using the same online platform, the structural conditions for common method variance are present. Given that the Teaching Beliefs scale's *AVE* (0.37) is also low, the possibility that residual method covariance has inflated the observed correlations—particularly between teaching beliefs and the other constructs—cannot be fully ruled out. Readers are therefore advised to treat the magnitude of the reported associations as upper-bound estimates. Future research should employ temporal separation of predictor and outcome measurements, informant-report designs, or the marker variable approach (Podsakoff et al., 2003) to provide stronger *Common Method Bias (CMB)* controls.

### 3.2 Measurement model

Results of the confirmatory factor analyses are presented in Table 1. The four-factor measurement model demonstrated good fit (χ^2^*/*df = 2.45, *CFI* = 0.93, *TLI* = 0.92, *RMSEA* = 0.051, *SRMR* = 0.044) and significantly outperformed the three-factor, two-factor, and single-factor alternatives.

**Table 1:** Comparison of measurement models and fit indices.

Model	*χ^2^*	*df*	*χ^2^/df*	*CFI*	*TLI*	*RMSEA*	*SRMR*
Four-factor model	4,127.58	1,684	2.45	0.93	0.92	0.051	0.044
Three-factor model^a^	5,831.24	1,687	3.46	0.87	0.86	0.066	0.068
Two-factor model^b^	7,245.67	1,689	4.29	0.82	0.81	0.076	0.082
Single-factor model	8,974.94	1,690	5.31	0.76	0.75	0.087	0.095

^a^Career adaptability and work engagement merged into one factor.

^b^Teaching beliefs and career adaptability merged into one factor, work engagement and wellbeing merged into one factor. The above model comparisons are exploratory structural comparisons and do not constitute a strictly nested model sequence; they are intended to demonstrate the superior discriminant validity of the four-factor structure.

For discriminant validity, average variance extracted (*AVE*), composite reliability (*CR*), and the heterotrait–monotrait ratio (*HTMT*) were calculated for each construct; results are presented in Table 2. The AVE values for career adaptability (*CA*) and work engagement (WE) were 0.49 and 0.58, respectively, meeting or near the AVE ≥ 0.50 criterion proposed by Fornell and Larcker (1981). The AVE for teaching beliefs (*TBS*) was 0.37 wiht CR = 0.84, and the AVE for GWB was 0.39, with *CR* = 0.90. Critically, all pairwise *HTMT* values ranged from 0.44 to 0.73, well below the 0.85 threshold recommended by Henseler et al. (2015), supporting adequate discriminant validity across all four constructs. For additional evidence of discriminant validity, the square root of each construct's AVE (√*AVE: TBS* = 0.61, *CA* = 0.70, *WE* = 0.76, *GWB* = 0.62) exceeded all off-diagonal *HTMT* values in the corresponding row and column (see Table 2), further supporting discriminant validity despite the below-threshold AVE values for TBS and *GWB*. Several methodological caveats regarding the below-threshold *AVE* values are warranted. First, although Fornell and Larcker (1981) and Huang et al. (2013), when AVE falls below 0.50, this exemption is conditional rather than universal, it does not fully resolve concerns about convergent validity when multiple constructs simultaneously exhibit below-threshold AVE. In the present study, both *TBS* (*AVE* = 0.37) and *GWB* (*AVE* = 0.39) exceed the 50% error threshold, meaning that on average, measurement error accounts for a greater proportion of item variance than the latent construct itself for these two scales. Readers should therefore treat the observed correlations involving TBS and *GWB* as potentially upwardly biased due to residual method variance, and interpret effect sizes with corresponding caution. Second, item-reduction analyses (e.g., removing items with standardized factor loadings below 0.60) were examined but resulted in substantial loss of content validity for these multidimensional scales; the full-scale structure was therefore retained to preserve construct representativeness. Future research should develop or validate shorter versions of these scales that achieve both content coverage and adequate convergent validity. Additionally, the *HTMT* value between career adaptability (*CA*) and work engagement (*WE*) was 0.727, which, while below the 0.85 threshold, is notably higher than other pairwise *HTMT* values (range: 0.44–0.60). This elevated value is consistent with the conceptual overlap between career adaptability—particularly its confidence and control dimensions—and work engagement's vigor and dedication components, both reflecting active investment in one's work. However, the four-factor CFA model significantly outperformed the three-factor model in which *CA* and *WE* were merged (Δχ^2^ = 1703.66, Δdf = 3, *p* < 0.001, Δ*CFI* = 0.06, Δ*RMSEA* = 0.15), providing sufficient statistical justification for treating them as distinct constructs in the present analyses. Theoretically, career adaptability is a meta-level psychosocial resource governing vocational coping and planning, whereas work engagement is a momentary psychological state of vigor, dedication, and absorption; they are conceptually distinguishable even when empirically correlated.

**Table 2:** Reliability, validity indices, and HTMT discriminant validity matrix for each construct.

Construct	Items	α	*CR*	*AVE*	*√AVE*	*HTMT_1_*	*HTMT_2_*	*HTMT_3_*	*HTMT_4_*
1. Teaching beliefs *(TBS)*	27	0.817	0.838	0.37	0.61	–			
2. Career adaptability *(CA)*	24	0.956	0.958	0.49	0.70	0.603	–		
3. Work engagement *(WE)*	17	0.959	0.959	0.58	0.76	0.447	0.727	–	
4. Wellbeing *(GWB)*	18	0.834	0.899	0.39	0.62	0.439	0.521	0.577	–

As supplementary evidence for convergent validity, composite reliability (*CR*) for *TBS* (0.838) and *GWB* (0.899) both substantially exceed the recommended 0.70 threshold (Hair et al., 2019), indicating that the variance attributable to the common factor remains adequate despite the below-threshold *AVE*. The below-threshold *AVE* for *TBS* and *GWB* appears to arise primarily from the retention of lower-loading boundary items that are necessary for content representativeness in these multidimensional constructs, rather than from systematic construct-irrelevant error inflation. Critically, simulation research demonstrates that *HTMT* constitutes a more reliable discriminant validity criterion than the Fornell–Larcker *AVE* comparison when constructs are multidimensional and item loadings are heterogeneous (Henseler et al., 2015)—conditions directly applicable to the present *TBS* (27 items, four dimensions) and *GWB* (18 items, mixed formats) scales. Because all *HTMT* values in this study fell well below 0.85 (range: 0.44–0.73), readers may reasonably conclude that the four constructs are empirically distinguishable. Nonetheless, the simultaneous below-threshold AVE for both the primary predictor (*TBS*) and the criterion variable (*GWB*) represents a substantive limitation: the measurement error accounts for a significant share of item variance for these two constructs, and the observed teaching beliefs–wellbeing correlation should be interpreted as a probable upper-bound estimate. Accordingly, the observed structural paths involving TBS and GWB should be interpreted as conservative estimates of the true relationships. This concern is carried forward as an explicit fifth limitation in Section 4.5.

### 3.3 Descriptive statistics and correlations

Descriptive statistics and correlations are presented in Table 3. Teaching beliefs, career adaptability, work engagement, and wellbeing were all significantly and positively intercorrelated (*r* = 0.376–0.696, *p* < 0.001), supporting Hypothesis 1.

**Table 3:** Descriptive statistics and correlation matrix (*N* = 561).

Variable	*M*	*SD*	1	2	3	4
1. Teaching beliefs	4.36	0.36	–			
2. Career adaptability	4.21	0.54	0.527^***^	–		
3. Work engagement	4.37	0.97	0.416^***^	0.696^***^	–	
4. Wellbeing	4.65	0.73	0.376^***^	0.447^***^	0.495^***^	–

^***^p < 0.001.

It is worth noting that the relatively high correlation between career adaptability (CA) and work engagement (WE); (*r* = 0.696) raises the question of potential construct overlap. As reported in Section 3.2, several empirical tests support their discriminant validity. The HTMT ratio between CA and WE well below the conservative 0.85 threshold (Henseler et al., 2015), and a competitive CFA model comparison confirmed that merging CA and WE into a single factor produced significantly worse fit than the hypothesized four-factor model. Theoretically, while both constructs involve the mobilization of psychological resources, they are conceptually distinct. The shared variance between these constructs reflects the inherent link between individual meta-resources and work-related states rather than construct redundancy. Future studies are encouraged to employ experience-sampling methods (ESM) to further decouple these constructs at the intra-individual level.

### 3.4 Moderated mediation model

The moderated mediation model was tested using PROCESS Model 7, with gender (female = 1, male = 0), age, teaching experience, educational attainment, and professional rank (standardized) as control variables. Regression results are presented in Table 4.

**Table 4:** Regression results for the moderated mediation model (*N* = 561).

Predictor	*B*	*SE*	*t*	*p*	*R^2^*	*ΔR^2^*	*f^2^*
Outcome: work engagement (*M*)					0.532	0.022^a^	0.047
Teaching beliefs (*X*)	0.091	0.035	2.617^**^	0.009			
Career adaptability (*W*)	0.680	0.035	19.425^***^	< 0.001			
*X* × *W*	0.123	0.024	5.110^***^	< 0.001			
Gender (female = 1)^b^	−0.149	0.067	−2.231^*^	0.026			
15.6-7.4,-1.3498pt Age (standardized)	0.274	0.066	4.184^***^	< 0.001			
Outcome: wellbeing (*Y*)					0.285		0.148^c^
Teaching beliefs (*X*)	0.197	0.040	4.900^***^	< 0.001			
Work engagement (*M*)	0.411	0.040	10.156^***^	< 0.001			

a: Δ*R*^2^ indicates the incremental variance explained by the interaction term. b: Gender was dummy-coded (1 = female, 0 = male). c: Effect sizes are reported as Cohen's *f*^2^. All continuous variables were standardized. Control variables included age, teaching experience, educational attainment, and professional rank. Interaction terms (*X* × *W*) represents the interaction term between Teaching Beliefs and Career Adaptability. Regression coefficients (*B*) and their associated standard errors (*SE*) were estimated using the PROCESS macro (Model 7) with 5,000 bootstrap resamples. Control variables, including educational attainment and professional rank, were included in the model but are not shown as their effects were non-significant (*p* > 0.05). ^*^p < 0.05, ^**^p < 0.01, ^***^p < 0.001.

The total effect of teaching beliefs on wellbeing was statistically significant (*c* = 0.367, *SE* = 0.040, *t* = 9.237, *p* < 0.001). After including the mediator, the direct effect remained significant (*c*′ = 0.197, *SE* = 0.040, *t* = 4.900, *p* < 0.001), indicating partial mediation by work engagement. Hypothesis 2 was supported.

In the regression equation predicting work engagement, teaching beliefs (*B* = 0.091, *SE* = 0.035, *t* = 2.617, *p* = 0.009), career adaptability (*B* = 0.680, *SE* = 0.035, *t* = 19.425, *p* < 0.001), and their interaction term (*B* = 0.123, *SE* = 0.024, *t* = 5.110, *p* < 0.001) were all significant, supporting Hypothesis 3. The incremental variance explained by the moderating effect was Δ*R*^2^ = 0.022, *Cohen's f*^2^ = 0.047 (small-to-medium effect). The model accounted for 53.2% of the variance in work engagement (*R*^2^ = 0.532).

### 3.5 Conditional indirect effects and index of moderated mediation

Conditional indirect effects estimated via bootstrapping (5,000 resamples) are presented in Table 5. At low levels of career adaptability (*M – 1SD*), the point estimate of the indirect effect was −0.013, 95% *CI* (−0.049, 0.024), which was not statistically significant. At the mean level (*M*), the indirect effect was 0.038 [CI: (0.006, 0.073)], which was significant. At high levels (*M* + *1SD*), the indirect effect was 0.088 [*CI*: (0.040, 0.142)], which was also significant. The index of moderated mediation was 0.051, 95% *CI* (0.022, 0.079), supporting Hypothesis 4. κ^2^ = 0.041 (small effect size).

**Table 5:** Conditional indirect effects (Bootstrap = 5,000).

Career adaptability level	Indirect effect	*SE*	95% CI		*p*
			LL	UL	
Low (*M* – 1SD)	−0.013	0.019	−0.049	0.024	0.493
Medium (*M*)	0.038	0.017	0.006	0.073	0.032
High (*M* + 1SD)	0.088	0.026	0.040	0.142	0.001
Index of moderated mediation	0.051	0.015	0.022	0.079	0.001

The *p* column reports exact bootstrap *p*-values (two-tailed) based on 5,000 resamples; 95% CIs are reported in the adjacent columns. *LL*, lower limit; *UL*, upper limit.

### 3.6 Simple slope analysis and Johnson–Neyman analysis

**Figure 2:**
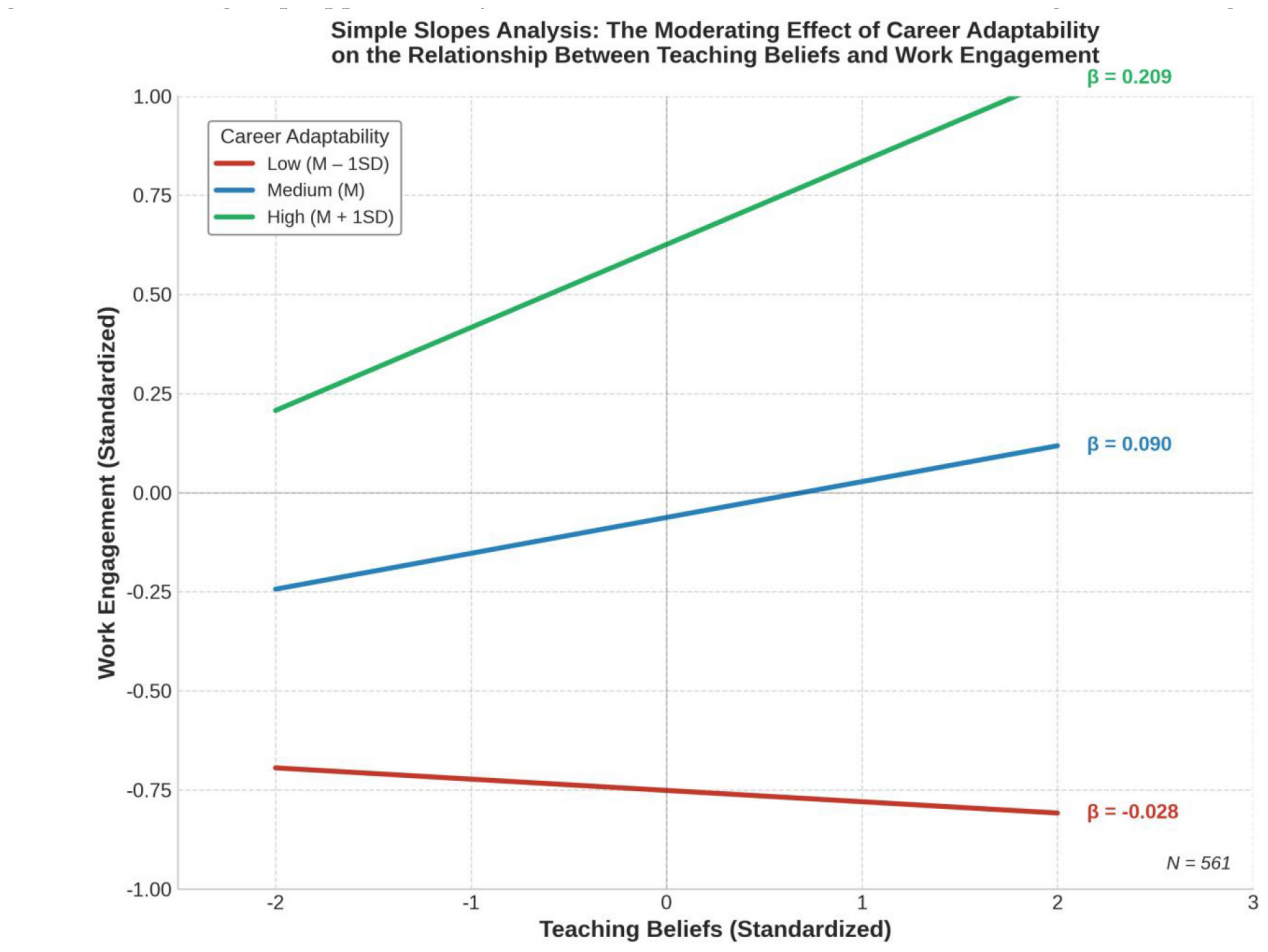
Moderating effect of career adaptability on the teaching beliefs–work engagement relationship (simple slope plot).

Simple slope analysis (Figure 2) revealed that, at low levels of career adaptability, the simple slope of teaching beliefs on work engagement was not significant (*B* = −0.032, *SE* = 0.040, *t* = −0.797, *p* = 0.426); at the mean level, it was significant (*B* = 0.091, *SE* = 0.035, *t* = 2.617, *p* = 0.009); and at high levels, it was further strengthened (*B* = 0.215, *SE* = 0.045, *t* = 4.808, *p* < 0.001).

Johnson–Neyman analysis indicated that the positive relationship between teaching beliefs and work engagement reached statistical significance (*p* < 0.05) when the standardized career adaptability score exceeded −0.189 (corresponding to a raw score of 4.11). In the present sample, approximately 56.3% of teachers (*n* ≈ 316) had raw career adaptability scores above this threshold, such that the beliefs–work engagement pathway was significant for this group; approximately 43.7% of teachers (*n* ≈ 245) fell within the region of non-significance.

## 4 Discussion

### 4.1 The relationship between teaching beliefs and teacher wellbeing

Teaching beliefs were significantly and positively correlated with teacher wellbeing (*r* = 0.376, *p* < 0.001), consistent with prior findings (Dreer, 2021; Friesen et al., 2023) and in line with the core tenet of social cognitive theory regarding a positive association between beliefs and emotional experience (Bandura, 1997). Teachers who hold positive teaching beliefs place greater value on their professional role and have stronger confidence in their teaching efficacy; these cognitive resources collectively furnish an important psychological basis for wellbeing. Within the Chinese context, despite the considerable work pressures faced by primary school teachers (Liao et al., 2023), positive teaching beliefs were still significantly and positively associated with wellbeing, echoing the findings of. It should be noted that, given the cross-sectional design of this study, the above association reflects statistical correlation rather than causal directionality; a reverse-causal hypothesis (i.e., that higher wellbeing promotes more positive teaching beliefs) cannot be ruled out within the present research design.

### 4.2 The mediating role of work engagement

Work engagement partially mediated the relationship between teaching beliefs and wellbeing, consistent with the motivational pathway hypothesis of the JD-R model (Bakker and Demerouti, 2017). Within the interpretive framework of cross-sectional associational data, the positive associations between teaching beliefs and work engagement, and between work engagement and wellbeing, together constitute an indirect beliefs–engagement–wellbeing pathway. These findings align with those of Hakanen et al. (2006) and Granziera et al. (2020). Notably, the direct effect (*c*′ = 0.197) remained significant and exceeded the indirect effect, suggesting that other associational pathways may also be relevant to the relationship between teaching beliefs and wellbeing. Future research could explore potential associational mediators such as emotion regulation and professional identity (Gross, 2015).

### 4.3 The moderating role of career adaptability and its theoretical implications

The central finding of this study is that career adaptability significantly moderated the cross-sectional relationship between teaching beliefs and work engagement (*B* = 0.123, *p* < 0.001). First, the present findings lend empirical support to the integrative analytical framework proposed in Section 1.3, which uses Career Construction Theory to address a specific explanatory gap within the JD-R model. The new theoretical mechanism identified is that career adaptability functions as a meta-resource that regulates the resource-conversion efficiency of the JD-R motivational pathway—that is, the degree to which personal resources (teaching beliefs) are successfully converted into motivational outcomes (work engagement). This mechanism is not native to the JD-R model, which does not specify the conditions under which personal resources translate into engagement, and Career Construction Theory uniquely supplies it (Coetzee and Stoltz, 2015). Teachers with high career adaptability can translate positive beliefs into purposeful engagement behaviors; by contrast, those with low career adaptability may encounter what we term a “belief–action gap,” possessing positive beliefs but lacking the meta-capacity to translate them into actual engagement (Collie and Martin, 2016). It should be noted that this conceptualization serves as a heuristic lens for interpreting the observed interaction pattern; its validity as a psychological construct requires direct empirical testing beyond the scope of the present cross-sectional data.

Second, this study identified significant conditional heterogeneity in the mediated effect. Notably, at low levels of career adaptability (*M – 1SD*), the point estimate of the indirect effect was −0.013, but *SE* = 0.019 and 95% *CI* = (−0.049, 0.024) spanned zero broadly, rendering the effect entirely non-significant statistically. The negative direction of this point estimate, although non-significant and therefore not interpretable as a reliable effect, warrants theoretical attention. One plausible mechanism is a “high-expectation, low-efficacy” frustration dynamic: teachers with strong positive beliefs but insufficient career adaptability (i.e., limited occupational concern, control, curiosity, and confidence) may experience heightened awareness of the gap between their pedagogical ideals and their actual behavioral capacity, generating a subjective sense of professional inadequacy and resource depletion (Hobfoll, 2001). Rather than translating beliefs into engaged action, these teachers may instead experience cognitive dissonance or motivational exhaustion—a pattern consistent with what Haenggli and Hirschi (2020) described as ineffective resource mobilization under low meta-resource conditions. This “believing but unable to act” predicament, if sustained, could ultimately attenuate rather than support work engagement, producing the observed negative (though unreliable) trend. This finding reveals that, against the backdrop of complex educational reform, relying solely on “belief-driven” approaches may lead to psychological resource depletion among teachers. From the perspective of Conservation of Resources Theory (Hobfoll, 2001), teaching beliefs constitute a guiding personal resource, whereas career adaptability is a “meta-resource” that governs the capacity to mobilize resources. For these teachers, although they may endorse progressive pedagogical ideas (high beliefs), when confronted with administrative burdens or the challenges of home-school communication, their insufficient occupational concern and sense of control (low adaptability) renders it difficult to translate belief intentions into sustained behavioral engagement (Haenggli and Hirschi, 2020), resulting in a predicament of “knowing but not doing.” Particularly during the period of digital educational transformation, the rapid pace of technological change may further amplify this conversion barrier, causing low-adaptability teachers to feel resource-depleted when pursuing their professional goals (Haenggli and Hirschi, 2023). Future research should directly test this frustration mechanism using longitudinal or experience-sampling designs to determine whether the negative trend at low adaptability is a reliable phenomenon or an artifact of the cross-sectional design.

The Johnson–Neyman analysis further indicated that the J–N transition point (raw score = 4.11) represents a statistical boundary at which the beliefs–work engagement slope crosses the significance threshold within this sample and on this particular scale; it should not be interpreted as a fixed psychological threshold with universal applicability. This boundary is both scale-dependent (i.e., specific to the 5-point CAAS metric used here) and sample-specific (i.e., derived from this particular sample of Guangdong primary school teachers). Accordingly, the finding that approximately 43.7% of teachers fell within the region of non-significance is offered as a sample-specific descriptive observation rather than a generalizable population estimate. With these caveats in mind, this pattern nonetheless alerts educational administrators to pay particular attention to teachers with relatively insufficient career adaptability when designing intervention programs, and to provide differentiated professional support. One additional noteworthy finding from the regression results (Table 4) deserves brief comment. Female teachers showed slightly lower work engagement than male teachers (*B* = −0.149, *p* = 0.026), a finding that may appear counterintuitive given that female teachers constituted 72.4% of the present sample and are generally considered to exhibit high occupational identification. Several explanations may account for this pattern. First, female primary school teachers in China frequently face compounded role demands—including domestic responsibilities, childcare, and the expectation of emotional labor with students and parents—that may generate work–family conflict (Sun et al., 2022; Zhong et al., 2025) and attenuate work engagement relative to their male colleagues (Sun et al., 2022). Second, structural constraints such as more limited access to leadership roles and professional title advancement for female teachers in the Chinese primary education system may reduce perceptions of occupational control and career prospect, which are key antecedents of engagement. Third, the higher absolute number of female respondents increases statistical power to detect small gender differences that may not be practically meaningful. The effect size (*B* = −0.149 on a standardized scale) is modest, and this finding should be interpreted with caution given the cross-sectional design. Future research should employ gender-sensitive designs to examine whether role conflict or structural inequity mediates gender differences in work engagement among Chinese primary school teachers.

### 4.4 Practical implications

Three practical implications are offered. First, greater attention should be paid to cultivating teachers' positive teaching beliefs. Professional development training, teaching research communities, and classroom observation and reflection activities can help primary school teachers develop constructivist, student-centered pedagogical orientations.

Second, particular focus should be placed on the group of teachers with low career adaptability. Priority should be given to enhancing their meta-resource levels through career adaptability workshops (to strengthen occupational concern and forward-planning capacities), mentoring programs (to build professional confidence and vocational skills), teaching innovation platforms (to stimulate occupational curiosity), and expanded curricular autonomy (to strengthen the sense of occupational control).

Third, a stratified and differentiated teacher development support system should be established to provide tailored professional development resources based on career adaptability levels. It should be acknowledged that implementing such interventions may encounter institutional resistance in school environments characterized by heavy administrative burdens and strong performance appraisal orientations, necessitating context-specific approaches.

### 4.5 Limitations and future directions

Four limitations of the present study warrant acknowledgment. First, all variables were measured at a single time point, precluding any directional causal inference. All associations reported herein, including the mediated and moderated pathways, are cross-sectional in nature and reflect statistical co-variation among variables at a single time point. Readers are explicitly cautioned against interpreting any finding in this study as evidence of causal directionality. For example, it remains possible that higher levels of wellbeing lead to more positive teaching beliefs, implying a reverse associative pathway (wellbeing → engagement → beliefs) that cannot be definitively excluded given the current study design. Similarly, the moderated mediation pathway identified here describes a conditional associational pattern rather than a causal mechanism. Future research should employ longitudinal designs (e.g., cross-lagged panel models) or randomized intervention experiments to establish the directionality of the relationships identified here and to provide stronger evidence for the causal mechanisms implied by the theoretical framework.

Second, sample representativeness: convenience sampling and online recruitment may have led to an overrepresentation of teachers with higher levels of digital literacy or work engagement; the urbanized context of Guangdong Province also limits the generalizability of findings to less-developed regions and rural schools. Furthermore, generalizability beyond the Chinese primary school context should not be assumed: the specific institutional pressures, professional title evaluation systems, and cultural norms surrounding teaching in China may render the magnitude and patterning of the reported associations non-transferable to Western or other non-Confucian educational contexts without replication. Cross-cultural replications using comparable measures are needed before international inferences are drawn.

Third, regarding the external validation evidence of a self-developed instrument: the Teaching Beliefs Scale (TBS; Wang et al., 2022) was originally authored by the correspondence author of this study. We have attempted to address potential concerns by ensuring the scale's psychometric robustness through multiple channels. The TBS was previously subjected to independent peer review and validated across both national (*N* = 681) and regional (*N* = 330) samples. For the current study, a Confirmatory Factor Analysis (CFA) conducted on an entirely new, independent sample (*N* = 561) demonstrated a strong model fit (χ^2^/df = 2.34, *CFI* = 0.91, *RMSEA* = 0.049), while its four-factor structure remains theoretically grounded in the multidimensional frameworks established by Pajares (1992) and Fives and Buehl (2012). Furthermore, the scale article has begun to see adoption by independent researchers (e.g., Zhang and Gan, 2005; Li et al., 2024), lending external weight to its construct validity. Nevertheless, despite these validation efforts and our explicit conflict-of-interest disclosure, the use of a self-authored tool introduces an inherent risk of allegiance bias. Consequently, we encourage future research to corroborate these results using measures developed by independent investigators or through multi-scale designs to verify cross-instrument convergent validity.

Fourth, measurement-level convergent validity: as reported in Section 3.2, both the Teaching Beliefs Scale and the GWB exhibited AVE values below the conventional 0.50 threshold (Fornell and Larcker, 1981). Critically, these two constructs encompass the primary predictor and the criterion variable in the present model, respectively. Although all pairwise *HTMT* discriminant validity values were satisfactory and *CR* was adequate for both scales, the simultaneous failure of the key predictor and criterion variable to meet the *AVE* criterion represents a substantive psychometric limitation that cannot be fully resolved by discriminant validity evidence alone. The below-threshold AVE implies that measurement error explains a greater proportion of item variance than the underlying construct does for these two scales, which may attenuate or inflate the observed teaching beliefs–wellbeing association. Consequently, the magnitude of reported correlations involving *TBS* and *GWB* should be interpreted as probable upper-bound estimates. Future research should apply item-response theory (IRT) models that explicitly partition construct variance from measurement error, or develop psychometrically refined short-forms of TBS and GWB that simultaneously achieve content representativeness and adequate AVE, so as to provide more precise effect-size estimates for the beliefs–engagement–wellbeing pathway.

## 5 Conclusions

Drawing on an integrative analytical framework drawing on the JD-R model and Career Construction Theory, the present study administered questionnaires to 561 primary school teachers and arrived at the following conclusions: (1) teaching beliefs are significantly and positively associated with teacher wellbeing; (2) work engagement partially mediates the cross-sectional relationship between teaching beliefs and wellbeing; (3) career adaptability moderates the first stage of the mediated pathway—under conditions of medium-to-high career adaptability, teaching beliefs are significantly and indirectly associated with wellbeing via work engagement, whereas this indirect effect is not statistically significant under conditions of low career adaptability; and (4) the index of moderated mediation is statistically significant, indicating that the strength of the indirect association increases with higher levels of career adaptability. All conclusions are based on cross-sectional data and reflect associational patterns among variables rather than causal relationships. These findings suggest that enhancing teacher wellbeing requires a dual approach encompassing both belief cultivation and adaptability development. For teachers with insufficient career adaptability, prioritizing the enhancement of meta-resource levels may serve as a critical condition for the beliefs–engagement–wellbeing associational pathway to become statistically detectable, providing a theoretical reference for teacher professional development interventions.

## Data Availability

The raw data supporting the conclusions of this article will be made available by the authors, without undue reservation.

## References

[B1] Bakker A. B. Demerouti E. (2017). Job demands–resources theory: taking stock and looking forward. J. Occup. Health Psychol. 22, 273–285. doi: 10.1037/ocp0000056 27732008

[B2] Bandura A. (1997). Self-efficacy: The Exercise of Control. New York, NY: Freeman.

[B3] Coetzee M. Stoltz E. (2015). Employees' satisfaction with retention factors: exploring the role of career adaptability. J. Vocat. Behav. 89, 83–91. doi: 10.1016/j.jvb.2015.04.012

[B4] Cohen J. (1988). Statistical Power Analysis for the Behavioral Sciences, (2nd edn.). Mahwah, NJ: Lawrence Erlbaum Associates.

[B5] Collie R. J. Martin A. J. (2016). Adaptability: an important capacity for effective teachers. Educ. Pract. Theory 38, 27–39. doi: 10.7459/ept/38.1.03

[B6] Dreer B. (2021). Teachers' well-being and job satisfaction: the important role of positive emotions in the workplace. Educ. Stud. 50, 61–77. doi: 10.1080/03055698.2021.1940872

[B7] Fazio A. F. (1977). A concurrent validational study of the nchs general well-being schedule Vital Health Stat. 73, 1–53. doi: 10.1037/e409022004-001 610049

[B8] Feng G. Shi K. Huang Q. Zhou J. (2024). The impact of work values on the professional development of primary and secondary school teachers: a moderated mediation model. PLoS ONE 19:e0310078. doi: 10.1371/journal.pone.0310078 39509437 PMC11542874

[B9] Fives H. Buehl M. M. (2012). “Spring cleaning for the messy construct of teachers' beliefs,” in APA Educational Psychology Handbook: Individual Differences and Cultural and Contextual Factors, eds. K. R. Harris, S. Graham, and T. Urdan (Washington, DC: American Psychological Association), vol. 2, 471–499. doi: 10.1037/13274-019

[B10] Fornell C. Larcker D. F. (1981). Evaluating structural equation models with unobservable variables and measurement error. J. Mark. Res. 18, 39–50. doi: 10.1177/002224378101800104

[B11] Friesen D. C. Shory U. Lamoureux C. (2023). The role of self-efficacy beliefs and inclusive education beliefs on teacher burnout. Soc. Sci. Humanit. Open 8:100599. doi: 10.1016/j.ssaho.2023.100599

[B12] Fritz M. S. MacKinnon D. P. (2007). Required sample size to detect the mediated effect. Psychol. Sci. 18, 233–239. doi: 10.1111/j.1467-9280.2007.01882.x 17444920 PMC2843527

[B13] Gao X. Zhou X. Leong F. T. L. (2024). Exploring occupational well-being profiles, outcomes, and predictors among Chinese teachers: a mixed-methods approach using latent profile and decision tree analysis. Appl. Psychol.: Health Well-Being 17:e12640. doi: 10.1111/aphw.12640 39686631

[B14] Granziera H. Collie R. Martin A. (2020). “Understanding teacher wellbeing through job demands–resources theory,” in Cultivating Teacher Resilience, ed. C. F. Mansfield (Dordrecht: Springer), 229–244. doi: 10.1007/978-981-15-5963-1_14

[B15] Gross J. J. (2015). Emotion regulation: current status and future prospects. Psychol. Inq. 26, 1–26. doi: 10.1080/1047840X.2014.940781

[B16] Haenggli M. Hirschi A. (2020). Career adaptability and career success in the context of a broader career resources framework. J. Vocat. Behav. 119:103414. doi: 10.1016/j.jvb.2020.103414

[B17] Haenggli M. Hirschi A. (2023). “Career adaptability,” in Career Psychology: Models, Concepts, and Counseling for Meaningful Employment, eds. W. B. Walsh, L. Y. Flores, P. J. Hartung, and F. T. L. Leong (Washington, DC: American Psychological Association), 213–233. doi: 10.1037/0000339-011

[B18] Hair J. F. Risher J. J. Sarstedt M. Ringle C. M. (2019). When to use and how to report results of PLS-SEM. Eur. Bus. Rev. 31, 2–24. doi: 10.1108/EBR-11-2018-0203

[B19] Hakanen J. J. Bakker A. B. Schaufeli W. B. (2006). Burnout and work engagement among teachers. J. Sch. Psychol 43, 495–513. doi: 10.1016/j.jsp.2005.11.001

[B20] Hascher T. Waber J. (2021). Teacher well-being: a systematic review of the research literature from 2000–2019. Educ. Res. Rev. 34:100411. doi: 10.1016/j.edurev.2021.100411

[B21] Hayes A. F. (2022). Introduction to Mediation, Moderation, and Conditional Process Analysis: A Regression-Based Approach (3rd ed.). New York, NY: Guilford Press.

[B22] Henseler J. Ringle C. M. Sarstedt M. (2015). A new criterion for assessing discriminant validity in variance-based structural equation modeling. J. Acad. Mark. Sci. 43, 115–135. doi: 10.1007/s11747-014-0403-8

[B23] Hobfoll S. E. (2001). The influence of culture, community, and the nested-self in the stress process: advancing conservation of resources theory. Appl. Psychol.: Int. Rev. 50, 337–421. doi: 10.1111/1464-0597.00062

[B24] Hou Z.-J. Leung S. A. Li X. Li X. Xu H. (2012). Career adapt-abilities scale–China form: construction and initial validation. J. Vocat. Behav. 80, 686–691. doi: 10.1016/j.jvb.2012.01.006

[B25] Huang C.-C. Wang Y.-M. Wu T.-W. Wang P.-A. (2013). An empirical analysis of the antecedents and performance consequences of using the Moodle platform. Int. J. Inf. Educ. Technol. 3, 217–221. doi: 10.7763/IJIET.2013.V3.267

[B26] IBM Corp. (2019). IBM SPSS Statistics for Windows, Version 26.0. Armonk, NY: IBM Corp.

[B27] Ji Y. Wang D. Riedl M. (2021). Analysis of the correlation between occupational stress and mental health of primary and secondary school teachers. Work 69, 599–611. doi: 10.3233/WOR-213502 34120938

[B28] Kun Á. Gadanecz P. (2022). Workplace happiness, well-being and their relationship with psychological capital: a study of Hungarian teachers. Curr. Psychol. 41, 185–199. doi: 10.1007/s12144-019-00550-0

[B29] Lesener T. Gusy B. Jochmann A. Wolter C. (2020). The drivers of work engagement: a meta-analytic review of longitudinal evidence. Work Stress 34, 259–278. doi: 10.1080/02678373.2019.1686440

[B30] Li K. Wijaya T. Chen X. Harahap M. (2024). Exploring the factors affecting elementary mathematics teachers' innovative behavior: an integration of social cognitive theory. Sci. Rep. 14:2581. doi: 10.1038/s41598-024-52604-4 38267501 PMC10808225

[B31] Liao J. Wang X.-Q. Wang X. (2023). The effect of work stress on the well-being of primary and secondary school teachers in China. Int. J. Environ. Res. Public Health 20:1154. doi: 10.3390/ijerph20021154 36673909 PMC9859342

[B32] Luján E. (2021). The beliefs of primary school teachers: a comparative analysis. Int. J. Instr. 14, 171–188. doi: 10.29333/iji.2021.14313a

[B33] Ministry of Education (2023). Ministry of Education of the People's Republic of China. National Education Development Statistical Bulletin 2022. Available online at: http://www.moe.gov.cn/jyb_sjzl/moe_560/2022/ (Accessed March 15, 2026).

[B34] Muthén L. K. Muthén B. O. (1998–2017). Mplus User's Guide (8th ed.). Los Angeles, CA: Muthén & Muthén.

[B35] Nalipay M. J. N. King R. B. Mordeno I. G. Wang H. (2022). Are good teachers born or made? Teachers who hold a growth mindset about their teaching ability have better well-being. Ed. Psychol. 42, 23–41. doi: 10.1080/01443410.2021.2001791

[B36] Nalipay M. J. N. Mordeno I. G. Semilla J. M. C. Frondozo C. E. (2019). Implicit beliefs about teaching ability, teacher emotions, and teaching satisfaction. Asia-Pac. Educ. Res. 28, 313–325. doi: 10.1007/s40299-019-00467-z

[B37] Pajares M. F. (1992). Teachers' beliefs and educational research: cleaning up a messy construct. Rev. Educ. Res. 62, 307–332. doi: 10.3102/00346543062003307

[B38] Podsakoff P. M. MacKenzie S. B. Lee J.-Y. Podsakoff N. P. (2003). Common method biases in behavioral research: a critical review of the literature and recommended remedies. J. Appl. Psychol. 88, 879–903. doi: 10.1037/0021-9010.88.5.879 14516251

[B39] Preacher K. J. Kelley K. (2011). Effect size measures for mediation models: quantitative strategies for communicating indirect effects. Psychol. Methods 16, 93–115. doi: 10.1037/a0022658 21500915

[B40] Preacher K. J. Rucker D. D. Hayes A. F. (2007). Addressing moderated mediation hypotheses: theory, methods, and prescriptions. Multivar. Behav. Res. 42, 185–227. doi: 10.1080/00273170701341316 26821081

[B41] Reppa G. Mousoulidou M. Tzovla E. Koundourou C. Christodoulou A. (2023). The impact of self-efficacy on the well-being of primary school teachers: a Greek-Cypriot study. Front. Psychol. 14:1223222. doi: 10.3389/fpsyg.2023.1223222 37928576 PMC10620719

[B42] Rudolph C. W. Lavigne K. N. Zacher H. (2017). Career adaptability: a meta-analysis of relationships with measures of adaptivity, adapting responses, and adaptation results. J. Vocat. Behav. 98, 17–34. doi: 10.1016/j.jvb.2016.09.002

[B43] Savickas M. L. (2013). “Career construction theory and practice,” in Career Development and Counseling: Putting Theory and Research to Work, (2nd edn.), eds. S. D. Brown and R. W. Lent (Hoboken, NJ: Wiley), 147–183.

[B44] Savickas M. L. Porfeli E. J. (2012). Career adapt-abilities scale: construction, reliability, and measurement equivalence across 13 countries. J. Vocat. Behav. 80, 661–673. doi: 10.1016/j.jvb.2012.01.011

[B45] Schaufeli W. B. Salanova M. González-Romá V. Bakker A. B. (2002). The measurement of engagement and burnout: a two-sample confirmatory factor analytic approach. J. Happiness Stud. 3, 71–92. doi: 10.1023/A:1015630930326

[B46] Shao Y. Jiang W. Zhu H. Zhang C. Xu W. (2025). The relationship between work stress and well-being among Chinese primary and secondary school teachers: the chain mediation of affective rumination and work engagement. BMC Psychol. 13:736. doi: 10.1186/s40359-025-02628-w 40181448 PMC11969784

[B47] Sun C. Feng X. Sun B. Li W. Zhong C. (2022). Teachers' professional identity and burnout among Chinese female school teachers: mediating roles of work engagement and psychological capital. Int. J. Environ. Res. Public Health. 19:13477. doi: 10.3390/ijerph192013477 36294054 PMC9603075

[B48] Voss T. Kunter M. (2020). Reality shock of beginning teachers? Changes in beginning teachers' emotional exhaustion and constructivist-oriented beliefs. J. Teach. Educ. 71, 292–306. doi: 10.1177/0022487119839700

[B49] Wang J. Zhu Y. Xu X. (2022). Development and preliminary application of a teaching beliefs scale for primary school teachers. J. Hanshan Norm. Univ. 43, 97–103. doi: 10.19986/j.cnki.1007-6883.2022.03.016

[B50] Zhang J. X. Gan Y. Q. (2005). A Chinese version of the Utrecht work engagement scale: an examination of reliability and validity. Chin. J. Clin. Psychol. 13, 268–270. doi: 10.16128/j.cnki.1005-3611.2005.03.005

[B51] Zhong S. Hu J. Xu H. (2025). The impact of family support on depression among primary and secondary school teachers in China: the serial mediating roles of subjective time pressure and work-family conflict. BMC Psychol. 13. doi: 10.1186/s40359-025-03543-w PMC12628968 41257779

